# Supplementary material for: Imaging thermal conductivity with nanoscale resolution using a scanning spin probe
Source: Nat Commun. 2015 Nov 20;6:8954. doi: 10.1038/ncomms9954 (PMC4673876; doi:10.1038/ncomms9954)
Supplement: Supplementary Information — Supplementary Figures 1-7, Supplementary Notes 1-2 and Supplementary References. [file ncomms9954-s1.pdf]

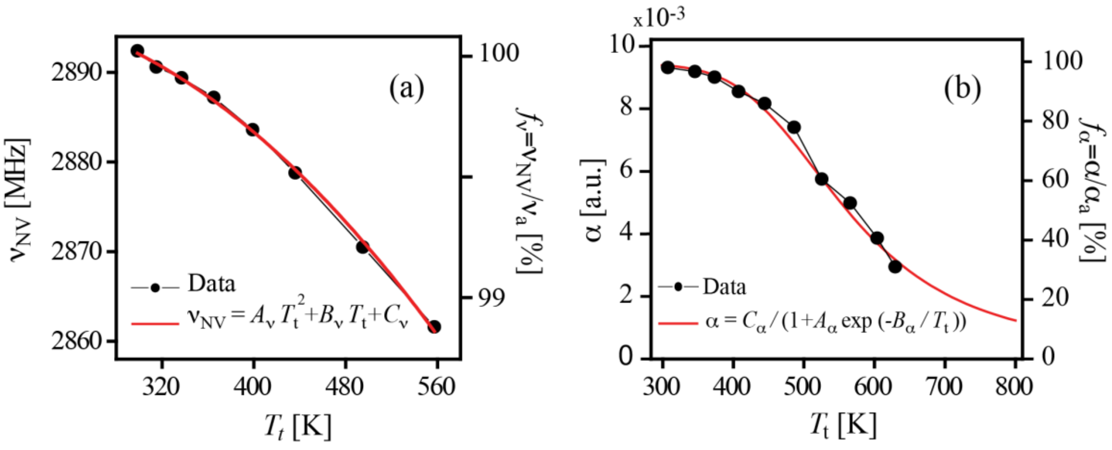

Supplementary Figure 1 | Thermal dependence of the NV spin resonance frequency and fluorescence. (a) Resonance frequency of the NV spin transition $m_S = 0 \leftrightarrow m_S = 1$ of the ground state triplet in the presence of a ~ 2 mT magnetic field as a function of the tip temperature T_t. Solid circles indicate data points; the continous red trace is a fit to the expression $\nu_{NV}(T_t) = A_\nu T_t^2 + B_\nu T_t + C_\nu$ with $A_\nu = -2.1 \times 10^{-4}$ MHz/K^2, $B_\nu = 6 \times 10^{-2}$ MHz/K and $C_\nu = 2893$ MHz. (b) Photon collection efficiency when the NV is in the bright state ($m_S = 0$) as a function of the tip temperature. Red squares are data points and the solid line is a fit to the expression $\alpha(T_t) = C_\alpha/(1 + A_\alpha \exp(-B_\alpha/T_t))$, with $A_\alpha = 600$, $B_\alpha = 3600$ K, and $C_\alpha = 9.4 \times 10^{-3}$.

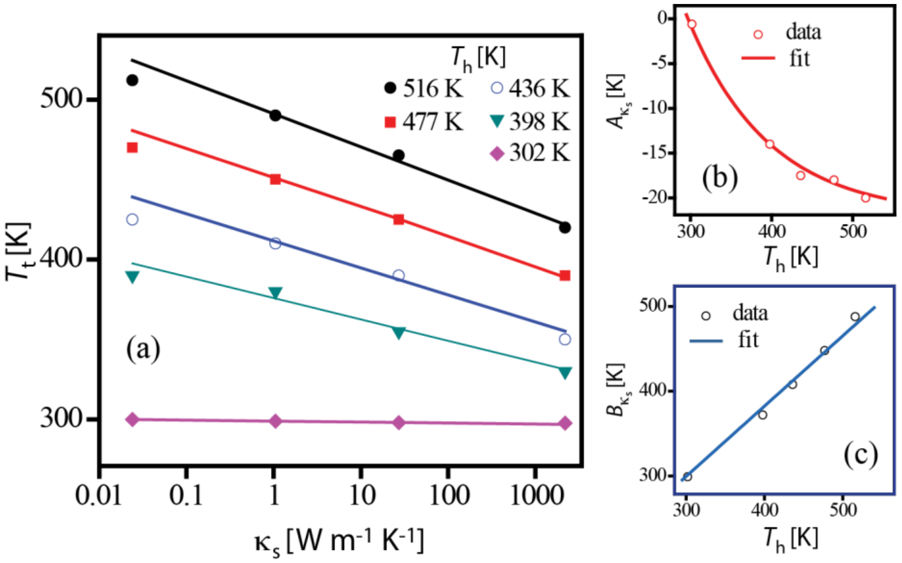

Supplementary Figure 2 | Tip temperature as a function of the substrate thermal conductivity. (a) Tip temperature T_t as a function of the substrate thermal conductivity κ_s for different heater temperatures T_h. Within the observed κ_s range we find the empirical relation $T_t(\kappa_s, T_h) = A_{\kappa_s}(T_h)\log(\kappa_s/\kappa_t(T_h))$, which we rewrite in the form $T_t = A_{\kappa_s}(T_h)\log\kappa_s + B_{\kappa_s}(T_h)$ with $B_{\kappa_s}(T_h) \equiv$ - $A_{\kappa_s}(T_h)\log\left(\kappa_t(T_h)\right)$. The solid lines correspond to fits to this latter expression. (b) A_{κ_s} as a function of T_h as determined from (a). The red solid trace indicates the fit to the expression $A_{\kappa_s}(T_h) = A_{\kappa_s}^{(0)}(1 - \exp(-(T_h - T_a)/\Delta T_A))$, with $A_{\kappa_s}^{(0)} = -22$ K, T_a=300 K, and $\Delta T_A = 100$ K. (c) Same as in (b) but for $B_{\kappa_s}(T_h)$. The solid straight line is a fit to the expression $B_{\kappa_s}(T_h) = B_{\kappa_s}^{(0)}(T_h - T_a)/\Delta T_B + T_a$, with $B_{\kappa_s}^{(0)} = 185$ K and $T_a = 300$ K; $\Delta T_B = 210$ K indicates the observed temperature range. In (b), (c) we express κ_s in units of Wm^{-1}K^{-1}.

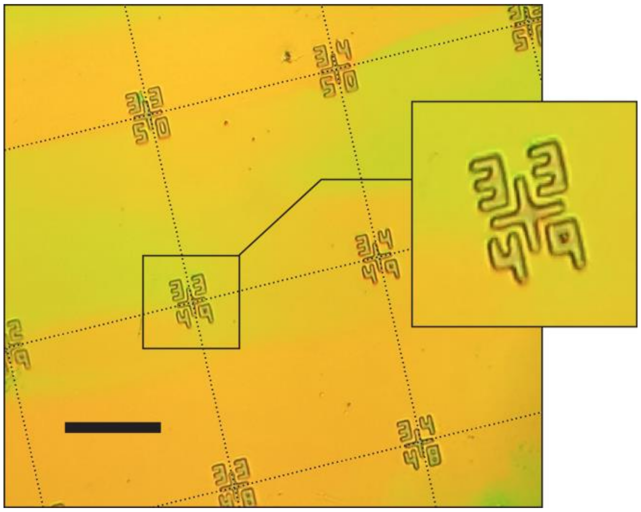

Supplementary Figure 3 | Optical image of the sample in Fig. 4 of the main text. The motif, a number-coded cross periodically repeated to form a square grid, is made out of 15 nm thick gold on single-crystal sapphire. The thick dark line in the lower left corresponds to 50 µm. The dashed line is a guide to the eye. The insert zooms on the structure studied here.

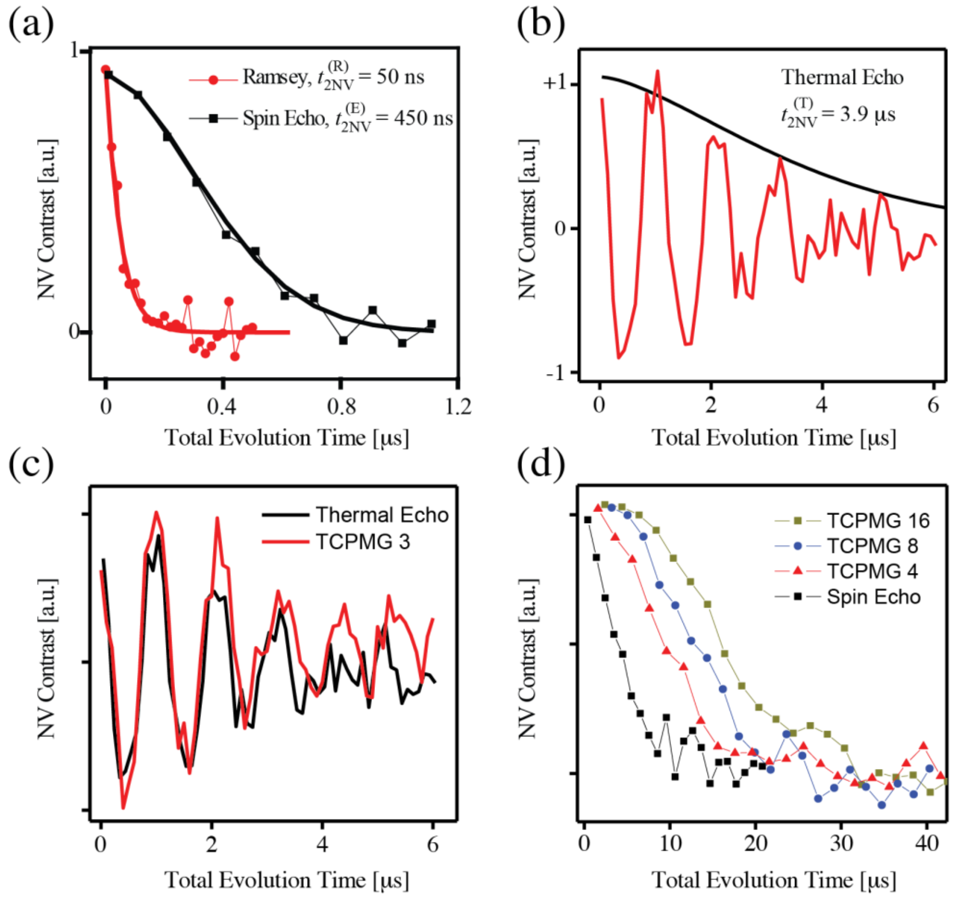

Supplementary Figure 4 | Characterization of the NV coherence lifetime. (a) Time-resolved, optically-detected magnetic resonance of the NV center in ND1 (see Fig. 2 of the main narrative). The solid red dots (black squares) indicate the response upon application of a 'Ramsey' ('spin echo') protocol. The red trace is an exponential fit $\exp\left(-t/t_{2NV}^{(R)}\right)$ to the Ramsey data with time constant $t_{2NV}^{(R)} = 50$ ns; the black trace is the corresponding fit to the spin-echo data using the expression $\exp\left(-\left(t/t_{2NV}^{(E)}\right)^2\right)$ with $t_{2NV}^{(E)} = 450$ ns. (b) NV signal upon application of a 'thermal echo' protocol as described in Ref. S3. (c) Application of a 'thermal' CPMG with 3 inversion pulses (TCPMG-3) as presented in Ref. S3. The thermal echo signal obtained in (b) is included for reference. (d) Amplitude of the NV echo signal after application of TCPMG sequences with a variable number of inversion pulses.

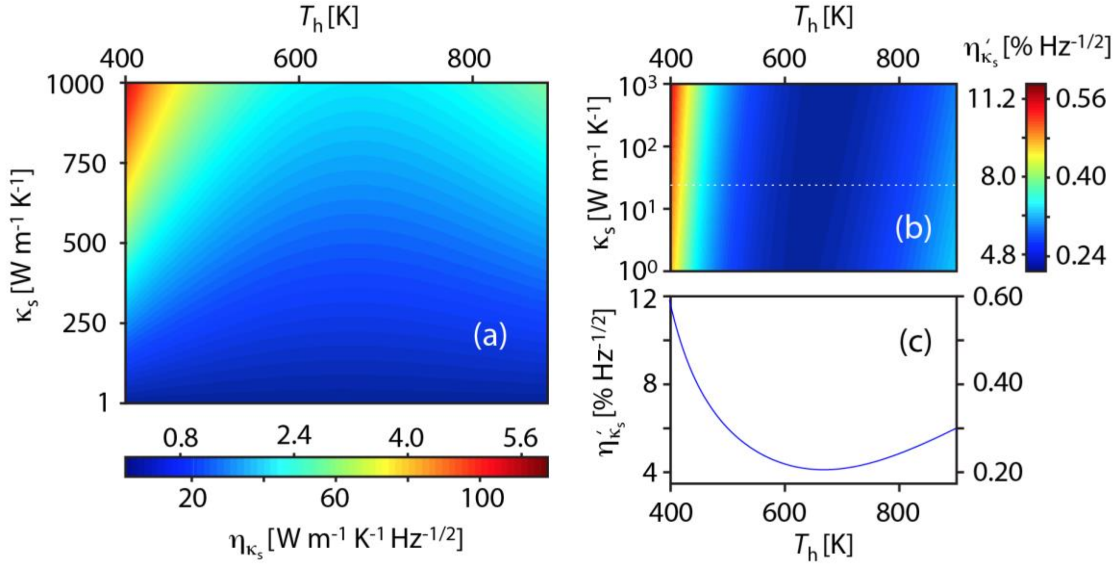

Supplementary Figure 5 | Determining the system sensitivity. (a) Calculated sensitivity to the substrate thermal conductivity η_{κ_s} as a function of the heater temperature T_h and the substrate thermal conductivity κ_s. The lower (upper) scale in the color bar corresponds to an NV coherence lifetime t_{2NV} of 500 ns (200 μs). (b) Relative sensitivity $\eta'_{\kappa_s} \equiv \eta_{\kappa_s}/\kappa_s$ as a function of T_h and κ_s. (c) Cross section of the plot in (b) at $\kappa_s = 30$ $\mathrm{Wm^{-1}K^{-1}}$. In (b) and (c) the left (right) η'_{κ_s} scale uses $t_{2NV} = 500$ ns ($t_{2NV} = 200$ μs). Best sensitivities are predicted for operating temperatures $T_h^{(opt)} \sim 620$ K.

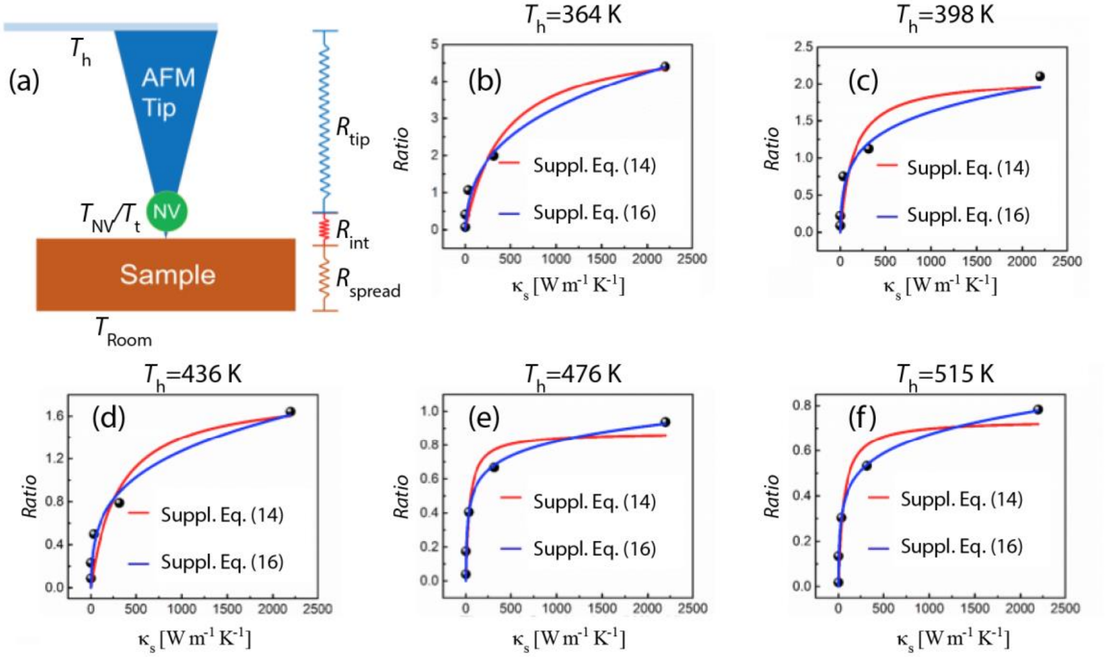

Supplementary Figure 6 | Modeling tip-sample thermal transport. (a) A cartoon of the one-dimensional thermal transport model system. Measured ratio (Eq. (S16)) vs. thermal conductivities for (b) T_h = 364 K, (c) T_h = 398 K, (d) T_h = 436 K, (e) T_h = 476 K and (f) T_h = 515 K. The red and blue curves are fitting curves with Eq. (S14) and Eq. (S16), respectively.

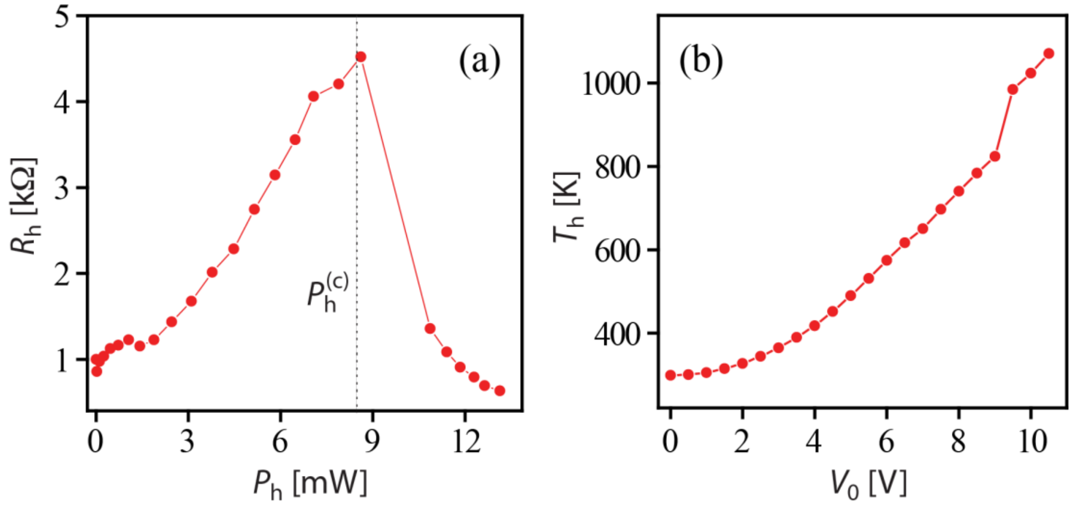

Supplementary Figure 7 | Characterization of the thermal cantilever. (a) Measured cantilever resistance R_h as a function of the dissipated power P_h. At $P_h^{(c)} = 8.6$ mW, thermal activation of the silicon carriers sharply reduces R_h. (b) From Eqs. (S2) and (S3), we determine T_h as a function of the applied voltage V_0.

Supplementary Note 1 | Sensitivity to the substrate thermal conductivity

The sensitivity to the substrate thermal conductivity is given by

$$\eta_{\kappa_s} \equiv \sqrt{t_{tot}}(\Delta\kappa_s)_{min} \,, \tag{1}$$

where t_{tot} is the total measurement time and $(\Delta\kappa_s)_{min}$ is the minimum detectable change of thermal conductivity. Assuming a shot-noise-limited signal, we recast Supplementary Eq. (1) as

$$\eta_{\kappa_s} = \sqrt{t_{tot}}(\delta S_{NV})_{shot}\left|\frac{dS_{NV}}{d\kappa_s}\right|^{-1} \,, \tag{2}$$

where $(\delta S_{NV})_{shot}$ is the shot noise and S_{NV} is the NV signal. From Supplementary Ref. 1, we know that

$$(\delta S_{NV})_{shot} \approx \sqrt{\frac{1}{2}(\alpha+\beta)N} \,, \tag{3}$$

where α (β) is the average number of photons collected during an individual measurement interval (~300 ns) when the NV spin is in the $m_S = 0$ $(m_S = 1)$ level of the ground state triplet, and N is the total number of repeats.

Unlike the experiments herein (where the full NV spectrum is recorded to determine $\Delta\nu_{NV}$, Fig. 2a), an optimized thermal sensing protocol would monitor the NV spin signal after a time t of coherent evolution[2,3]. In this case, the NV signal is given by

$$S_{NV}(\nu_{NV}, t_{2NV}) \approx \frac{(\alpha-\beta)}{2}N\cos(2\pi t\,\Delta\nu_{NV})\; e^{-\left(\frac{t}{t_{2NV}}\right)^m} + \frac{(\alpha+\beta)N}{2}, \tag{4}$$

$\Delta\nu_{NV} \equiv \nu_{NV} - \nu_{ref}$ is the NV resonance frequency shift relative to a reference, t_{2NV} is the NV spin coherence lifetime, and m is an integer in the range from 1 to 4. Since α, β, and ν_{NV} are functions of the nanocrystal temperature $T_t(\kappa_s, T_h)$, Supplementary Eq. (2) takes the form

$$\eta_{\kappa_s} = \sqrt{t_{\mathrm{tot}}}(\delta S_{\mathrm{NV}})_{\mathrm{shot}} \left| \left(\frac{dS_{\mathrm{NV}}}{dT_{\mathrm{t}}}\right) \left(\frac{dT_{\mathrm{t}}}{d\kappa_{\mathrm{s}}}\right) \right|^{-1} , \tag{5}$$

where

$$\left|\frac{dS_{\mathrm{NV}}}{dT_{\mathrm{t}}}\right| \cong \frac{\alpha N}{6}\, 2\pi t\, exp\left(-\left(\frac{t}{t_{2\mathrm{NV}}}\right)^m\right)\left(\frac{d\nu_{\mathrm{NV}}}{dT_{\mathrm{t}}}\right) + 0.85N\left(\frac{d\alpha}{dT_{\mathrm{t}}}\right) . \tag{6}$$

In deriving Supplementary Eq. (6), we are neglecting the temperature dependence of $t_{2\mathrm{NV}}$, comparatively week within the observed range, and we use $\beta = 0.7\alpha$.[4] Choosing the near-optimum evolution interval $t_{\mathrm{opt}} = t_{2\mathrm{NV}}/(m)^{1/m}$, and using Supplementary Eqs. (3) and (6) we recast Supplementary Eq. (5) as

$$\eta_{\kappa_s} \approx \sqrt{\frac{0.85\,\alpha\,\left(t_{2\mathrm{NV}}/\sqrt{2} + t_{\mathrm{d}}\right)}{\alpha_{\mathrm{a}}^2}} \left| \left(0.5\, f_\alpha\, t_{2\mathrm{NV}}\, \nu_a \frac{df_\nu}{dT_{\mathrm{t}}} + 0.85 \frac{df_\alpha}{dT_{\mathrm{t}}}\right)\left(\frac{dT_{\mathrm{t}}}{d\kappa_{\mathrm{s}}}\right) \right|^{-1} . \tag{7}$$

In Supplementary Eq. (7) $f_\nu \equiv \nu_{\mathrm{NV}}(T_{\mathrm{t}})/\nu_a$ and $f_\alpha \equiv \alpha(T_{\mathrm{t}})/\alpha_a$ denote the fractional NV resonance frequency and photon collection efficiency, respectively, where $\nu_{\mathrm{a}} = 2.89$ GHz and $\alpha_{\mathrm{a}} = 10^{-2}$ are the corresponding values at room temperature; we also assume $m = 2$ and $t_{\mathrm{tot}} = N\left(t_{\mathrm{opt}} + t_{\mathrm{d}}\right)$, where $t_{\mathrm{d}} \approx 300$ ns is the photon detection time. From the data in Supplementary Fig. 1a, we write

$$\nu_{\mathrm{NV}}(T_{\mathrm{t}}) = A_\nu T_{\mathrm{t}}^2 + B_\nu T_{\mathrm{t}} + C_\nu , \tag{8}$$

with $A_\nu = -2.1 \times 10^{-4}$ MHz/K^2, $B_\nu = 6 \times 10^{-2}$ MHz/K, and $C_\nu = 2893$ MHz. Following Supplementary Ref. (4), we describe $\alpha(T_{\mathrm{t}})$ via the formula

$$\alpha(T_{\mathrm{t}}) = \frac{C_\alpha}{1 + A_\alpha \exp(-B_\alpha/T_{\mathrm{t}})} , \tag{9}$$

with the parameters $A_\alpha = 600$, $B_\alpha = 3600$ K, and $C_\alpha = 9.4 \times 10^{-3}$ determined from a fit to our experimental data (Supplementary Fig. 1b). Using the Ramsey coherence lifetime $t_{2\mathrm{NV}}^{(\mathrm{R})} \cong 50$ ns (Supplementary Fig. 4), we find $0.5\, f_\alpha\, t_{2\mathrm{NV}}\, \nu_a \frac{df_\nu}{dT_{\mathrm{t}}} \sim 0.85 \frac{df_\alpha}{dT_{\mathrm{t}}} \sim 2 \times$

10^3 K^{-1} at $T_\mathrm{t} = 500$ K, implying that contributions to η_{κ_s} originating from a thermally-induced resonance shift or a fluorescence change are comparable for short lived NV spins evolving under the simplest sensing protocol (see Supplementary Eq. (7)). This is not the case, however, if echo sequences are exploited to extend the NV spin coherence lifetime. In the regime $t_{2\mathrm{NV}} \gtrsim 500$ ns we find $t_{2\mathrm{NV}}\, \nu_a \frac{df_\nu}{dT_\mathrm{t}} \gg \frac{df_\alpha}{dT_\mathrm{t}}$ and Supplementary Eq. (7) simplifies to

$$\eta_{\kappa_\mathrm{s}} \sim \frac{2}{\sqrt{\alpha(T_\mathrm{t})\, t_{2\mathrm{NV}}}} \left| \left(\frac{d\nu_\mathrm{NV}}{dT_\mathrm{t}}\right) \left(\frac{dT_\mathrm{t}}{d\kappa_\mathrm{s}}\right) \right|^{-1}, \qquad (10)$$

where we ignore contributions of order $t_\mathrm{d}/t_{2\mathrm{NV}}$.

In general, the tip temperature T_t is a function of the substrate thermal conductivity and the heater temperature T_h. From our experiments, we find

$$T_\mathrm{t}(\kappa_\mathrm{s}, T_\mathrm{h}) \sim A_{\kappa_\mathrm{s}}(T_\mathrm{h}) \log \kappa_\mathrm{s} + B_{\kappa_\mathrm{s}}(T_\mathrm{h}) \,, \qquad (11)$$

where A_{κ_s} and B_{κ_s} are empirical, T_h-dependent parameters (see Supplementary Fig. 2). Therefore, combining Supplementary Eqs. (8)-(11), we rewrite η_{κ_s} as

$$\eta_{\kappa_\mathrm{s}}(\kappa_\mathrm{s}, T_\mathrm{h}) \sim \frac{4}{\sqrt{t_{2\mathrm{NV}}}} \frac{\kappa_\mathrm{s}\, (1 + A_\alpha \exp(-B_\alpha / T_\mathrm{t}(\kappa_\mathrm{s}, T_\mathrm{h})))^{1/2}}{A_{\kappa_\mathrm{s}}(T_\mathrm{h})\, C_\alpha^{1/2}\, (2A_\nu T_\mathrm{t}(\kappa_\mathrm{s}, T_\mathrm{h}) + B_\nu)} \,, \qquad (12)$$

where $T_\mathrm{t}(\kappa_\mathrm{s}, T_\mathrm{h})$ is given by Supplementary Eq. (11).

A plot of the calculated sensitivity as determined from Supplementary Eq. (12) is presented in Supplementary Fig. 5a; for comparison we use two alternate scales corresponding to $t_{2\mathrm{NV}} = 500$ ns (representative of our present experimental conditions, see Supplementary Fig. 4), and $t'_{2\mathrm{NV}} = 200$ μs (possible in high-purity diamond nanocrystals[5]). Supplementary Fig. 5b displays the relative thermal sensitivity $\eta'_{\kappa_\mathrm{s}} \equiv \eta_{\kappa_\mathrm{s}}/\kappa_\mathrm{s}$, arguably more informative in situations where the substrate thermal conductivity

does not exhibit large changes (e.g., systems of uniform composition but varying local phase or crystallographic orientation). Optimum sensitivity is predicted at $T_{\mathrm{h}}^{(opt)} \sim 650$ K, the tradeoff point between the larger frequency shift at higher heater temperature (Supplementary Fig. 1a) and the concomitant reduction of NV fluorescence (Supplementary Fig. 1b). At this heater temperature, $\eta'_{\kappa_s}\big|_{500\ ns} \sim 4$ % $\mathrm{Hz}^{-1/2}$ for an NV coherence lifetime $t_{2\mathrm{NV}} = 500$ ns, the present experimental conditions. For NV centers in ultra-pure nanocrystals undergoing dynamical decoupling, lifetimes of up to 200 μs have been demonstrated, leading to a projected relative sensitivity of $\eta'_{\kappa_s}\big|_{200\ \mu s} \sim 0.2$ % $\mathrm{Hz}^{-1/2}$. In the same vein, improved sensitivity can be attained with the use of nanoparticles hosting multiple NVs[6] and/or by improved photon collection efficiency. For example, a nanoparticle hosting 500 NVs would lower η_{κ_s} by a factor $500^{1/2} \sim 20$. Finally, we note that in Supplementary Eq. (11) A_{κ_s} and B_{κ_s} are also functions of the heater material, implying that different sensitivities are to be expected for cantilevers of different composition.

Supplementary Note 2 | Tip-Sample Thermal Transport and Quantitative Measurements

To quantitatively understand the thermal transport and temperature gradient of the tip – sample system, we6use a one-dimensional thermal transport model as shown in Supplementary Fig. 7a. This 1D system consists of three parts in series: The AFM tip, the tip-sample interface and the sample substrate. The corresponding thermal resistances are denoted as R_{tip}, R_{int} and R_{spread}, respectively. Based on Fourier's law in 1D systems, the

temperature difference between any two points is proportional to the thermal resistance between these two points (similar to Ohm's Law), thus yielding the relation:

$$\frac{T_{\mathrm{h}} - T_{\mathrm{t}}}{T_{\mathrm{t}} - T_{\mathrm{room}}} = \frac{R_{\mathrm{tip}}}{R_{\mathrm{int}} + R_{\mathrm{spread}}} \tag{13}$$

Note that all parameters on the left side of Supplementary Eq. (13) can be determined by our experiments. In Supplementary Eq. (13), R_{tip} can be regarded as sample-independent constant as long as all the measurements are done with the same AFM tip. $R_{\mathrm{spread}} \sim 1/4a\kappa_{\mathrm{s}}$, where a is the contact radius (not tip radius) and κ_{s} is the sample thermal conductivity[7]. In Supplementary Eq. (13) the effective interfacial thermal resistance R_{int} is difficult to determine. Fourier's theory predicts that the heat transfer is a diffusive process at the interface between two materials. However, this classical theory fails when the length scale is smaller than the mean free path of the energy carriers, which can be several hundred nanometers for crystalline materials. A previous study has shown that in the case of nanometer scale contacts, the heat flow is "ballistic" rather than "diffusive"[8]. Therefore, the determination/measurement of the interfacial thermal resistance is challenging for thermal transport studies at the nanoscale. Supplementary Figs. 6b-6f show the measured $Ratio \equiv \frac{T_{\mathrm{h}} - T_{\mathrm{t}}}{T_{\mathrm{t}} - T_{\mathrm{room}}}$ vs. thermal conductivities for different samples at different cantilever nominal temperatures T_{h}. If we consider R_{int} as a constant for all the materials, we can fit the data points with the following function:

$$\frac{T_{\mathrm{h}} - T_{\mathrm{t}}}{T_{\mathrm{t}} - T_{\mathrm{room}}} = \frac{R_{\mathrm{tip}}}{R_{\mathrm{int}} + 1/4a\kappa_{\mathrm{s}}} \tag{14}$$

The contact radius a is approximately 10 nm. The resulting fitting curves (red) to the experimental data are shown in Supplementary Fig. 6 with the corresponding raw data

points. The fitting is not ideal, but it shows that Supplementary Eq. (14) can capture part of the thermal transport physics of our system. Furthermore, it provides an approximate relationship between the measured ratio and the thermal conductivities of different materials, which can be used to estimate the thermal conductivity of an unknown material. Also worth noting is that the assumption in Supplementary Eq. (14) that R_{int} is constant for different materials is clearly a crude approximation. In general, R_{int} depends on the material properties (particularly on its thermal conductivity) so a reasonable hypothesis is

$$R_{\mathrm{int}} = C/(\kappa_{\mathrm{s}})^{n} \qquad (15)$$

where C and n are both constants. Combining Supplementary Eqs. (14) and (15), we have:

$$\frac{T_{\mathrm{h}} - T_{\mathrm{t}}}{T_{\mathrm{t}} - T_{\mathrm{room}}} = \frac{R_{\mathrm{tip}}}{C/(\kappa_{\mathrm{s}})^{n} + 1/4a\kappa_{\mathrm{s}}} \qquad (16)$$

The fitting curves (blue) using Supplementary Eq. (16) are also shown in Supplementary Fig. 6 along with raw data points and the original fitting curves from Supplementary Eq. (14). Not only the agreement is better but, more remarkably, the exponent n has a very small range 0.2 to 0.3 for the different temperatures and materials, with n_{average} ~ 0.25. The fitting also gives $R_{\mathrm{tip}} = 5\times10^{6}$ K/W which is in good agreement with previous results. Supplementary Eq. (16) with n ~ 0.25, $R_{\mathrm{tip}} = 5\times10^{6}$ K/W and C = 2×10^{7} is therefore a relationship which can be used with good accuracy to determine the thermal conductivity of an unknown material at the nanoscale from a measured temperature ratio (we assume SI units for κ_{s}).

Supplementary References

[1] Meriles, C.A. *et al.* Imaging mesoscopic nuclear spin noise with a diamond magnetometer, *J. Chem. Phys.* **133**, 124105 (2010).

[2] Maze, J.R. *et al.* Nanoscale magnetic sensing with an individual electronic spin in diamond, *Nature* **455**, 644–647 (2008).

[3] Toyli, D.M. *et al.* Fluorescence thermometry enhanced by coherence of single spins in diamond, *Proc. Natl. Acad. Sci. USA* **110**, 8417–8421 (2013).

[4] Toyli, D.M. *et al.* Measurement and control of single nitrogen-vacancy center spins above 600 K, *Phys. Rev. X* **2**, 031001 (2012).

[5] Trusheim, M.E. *et al.* Scalable fabrication of high purity diamond nanocrystals with long-spin-coherence nitrogen vacancy centers, *Nano Letters* **14**, 32–36 (2014).

[6] Kucsko, G. et al. Nanometre-scale thermometry in a living cell, *Nature* **500**, 54–58 (2013).

[7] Yovanovich, M.M., Culham, J.R. & Teertstra, P. Analytical modeling of spreading resistance in flux tubes, half spaces, and compound disks, *Components, Packaging, and Manufacturing Technology, Part A, IEEE Transactions on* **21**, 168–176 (1998).

[8] Siemens, M.E. *et al.* Quasi-ballistic thermal transport from nanoscale interfaces observed using ultrafast coherent soft X-ray beams, *Nature Mater.* **9**, 26–30 (2010).
